# Gravidic Intrahepatic Cholestasis With Concurrent COVID-19 Infection

**DOI:** 10.7759/cureus.29326

**Published:** 2022-09-19

**Authors:** Nidhi Makhija, Surekha Tayade, Utkarsh Thatere

**Affiliations:** 1 Department of Obstetrics and Gynecology, Jawaharlal Nehru Medical College, Datta Meghe Institute of Medical Sciences (Deemed to be University), Wardha, IND; 2 Department of Medicine, Jawaharlal Nehru Medical College, Datta Meghe Institute of Medical Sciences (Deemed to be University), Wardha, IND

**Keywords:** intrahepatic cholestasis, pregnancy, cholestatic jaundice, pruritis, covid-19

## Abstract

The most usual pregnancy-specific liver condition that commonly exhibits in the third trimester is intrahepatic cholestasis (IHC). Maternal non-pruritic rash and jaundice are clinical signs; and abnormal liver function tests, especially elevated blood bile acids, are the laboratory findings. Pregnancy-related IHC is linked to a higher risk of unfavorable perinatal consequences including stillbirth, meconium-stained amniotic fluid, and spontaneous premature delivery especially when combined with COVID-19 infection. The treatment for it typically involves ursodeoxycholic acid. There is mounting evidence that IHC during pregnancy may have long-term effects on the health of both the mother and the fetus. Therefore, to have a better understanding of the etiology, management and consequences on maternal and fetal wellbeing, with concurrent COVID-19 infection; here is a case of a 25-year-old second gravida with IHC with concurrent COVID-19 infection in the discussion.

## Introduction

Intrahepatic cholestasis of pregnancy (ICP) is a kind of cholestatic syndrome that develops in the second or third trimester of pregnancy and is characterized by pruritus, increased bile acid and blood aminotransferase levels. Symptoms spontaneously disappear two to three weeks after birth. The first case of unexplained pruritis with overt jaundice was noted in a pregnant lady in her third trimester in 1883 [1]. The symptoms lingered till birth and then disappeared on their own. The main clinical sign of ICP is pruritus. Pruritus can range from mild for some to severe with debilitating consequences for other patients. Pruritis occurs without any associated skin lesions that are worse in the evening and involves the palms of the hands and soles of the feet. It often manifests in the third trimester, usually or mostly after 30 weeks of gestation; however, there have been a few uncommon cases reported where it appeared as early as six to 10 weeks.

In 10% to 15% of instances, there is mild jaundice with just mildly increased conjugated bilirubin blood levels. Jaundice usually appears 1-4 weeks after pruritis and rarely as the first sign of pruritis. Women with a history of ICP (as well as first-degree relatives) had higher rates of gallstone development and cholecystitis than the general population (rate ratio: 3.7) [2,3]. However, if associated should prompt further evaluation for coexisting conditions like cholelithiasis, hepatitis, etc.

Elevated serum bile acids and aminotransferase activity are the primary metabolic changes. Serum total bilirubin levels that may increase 10-100 times over the normal limit and maternal fasting bile acid levels more than 40 μmol/L with a normal value of up to (11.0 μmol/L) were linked to a higher incidence of fetal abnormalities. The prognosis for the mother is favorable, and following birth, symptoms disappear quickly while serum liver tests return to normal. The development of pruritus in late pregnancy may be related to various chronic liver illnesses like primary sclerosing cholangitis, chronic hepatitis C, or primary biliary cirrhosis. ICP, especially when combined with fasting blood bile acid levels > 40 mol/L, increases the risk of preterm delivery (up to 19%-60%), meconium staining of amniotic fluid (up to 27%), fetal distress (up to 22%-41%), fetal bradycardia (up to 14%), and fetal death (up to 0.4%-4.1%) [4]. In a recent investigation, the intensity of pruritus upon diagnosis and fasting serum bile acid levels were independent predictors of preterm birth.

COVID-19 associated when combined with intrahepatic cholestasis adds up to the deranged liver function tests [5]. Henceforth, we will get into the case in detail involving the management and treatment of a second gravida with ICP and COVID-19 infection.

## Case presentation

A 25-year-old second gravida at 36 weeks of gestation presented in an emergency unit of a tertiary care center with complaints of non-resolving jaundice and itching for the past two weeks and a history of fever for the past two days. She had a pale-colored stool, generalized intense itching, dark-colored urine, and yellowish discoloration of her sclera, nail tips, and cheeks. She had also been experiencing sleep deprivation for a week, due to intense itching all over the body. The clinical examination revealed that she was of average build, and pale in appearance with evident itching, not related to subsequent skin lesions. She was lethargic. While her vital signs stayed within normal range. Her COVID-19 antigen test was performed in the emergency department, and she tested positive for COVID-19. Her saturation on room air was maintained at 99% and was categorized as a Mild case based on her saturation and symptoms and kept in isolation.

An ultrasound scan showed a live single intrauterine fetus at about 36 weeks gestation, with an estimated fetal weight of 2827+/- 300g, an inadequate amniotic fluid measuring 7.2 cm, and a normal Doppler flow. Bile salts and pigments were detected in the urine sample. Serum indicators were still increased, with notable increases in S. bilirubin level of 8.1 mg/dL with a normal range of 0.1-1.2 mg/dL, serum glutamic-oxaloacetic transaminase (SGOT) of 46 IU/L with normal range of 10-40 IU/L, alkaline phosphatase (ALP) of 141 IU/L with normal range of 24-147 IU/L, Sr. albumin of 3.1 g/dL with normal range of 2.4-4 g/dL, prothrombin time of 14.0 with normal control of 11.0 seconds, activated partial thromboplastin time (aPTT) of 46.6 with normal control of 30.0 seconds, and hemoglobin of 11.0 g/dL with normal range of 11.0 g/dL, and peripheral smear result suggested normocytic normochromic anemia. The possibility of pre-eclampsia was ruled out. The patient was tested for hepatitis A and B on gastroenterology advice which was both negative and she was commenced on 300 mg three times daily (TID) ursodeoxycholic acid.

She was scheduled for induction of labor with vaginal misoprostol at 37 weeks of gestation. Her first stage of labor lasted for five hours and then she delivered vaginally under all aseptic precautions, the fetal outcome was good. Labour was managed according to protocol. The baby's APGAR scores at 1, 5, and 10 minutes after birth were seven, eight, and nine, respectively. The baby was screened for hepatitis profile. At birth, no malformations were found. She was administered injection of Vitamin K post-delivery prophylactically due to delayed reabsorption of vitamin K in cases of intrahepatic cholestasis during pregnancy. The trend of serum bilirubin values is shown in Table 1.

**Table 1:** Liver Function Test Values

Date	Serum Total bilirubin (mg/dL)	Conjugated bilirubin (mg/dL)	Serum Glutamic-Oxaloacetic Transaminase (IU/L)	Alkaline Phosphatase (IU/L)	Serum Albumin (g/dL)
23.08.2021	8.1	6.8	46	141	3.1
26.08.2021	6.5	5.6	55	168	3.0
29.08.2021	6.1	5.2	54	160	3.1
01.09.2021	5.2	4.4	38	140	2.9
04.09.2021	3.3	2.8	37	110	3.2
06.09.2021	2.7	0.5	40	105	3.3

**Figure 1:**
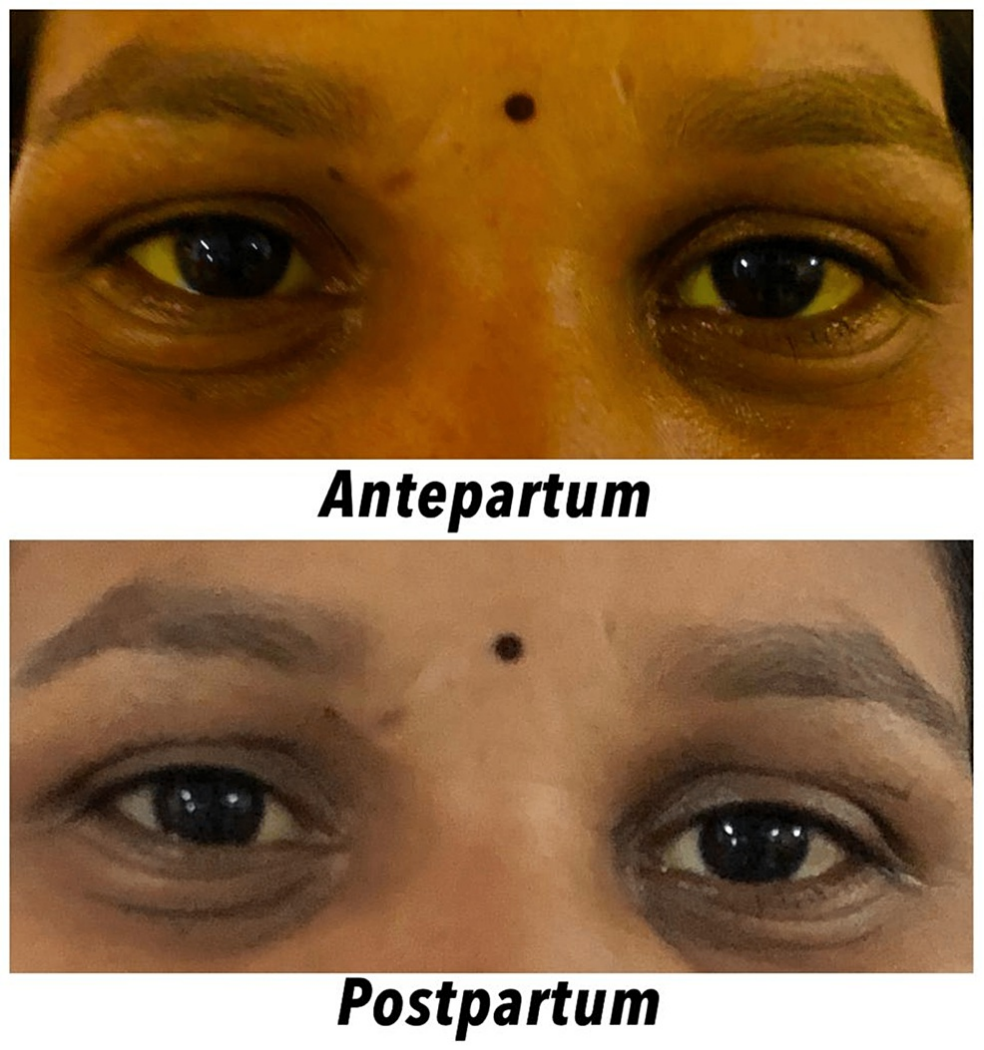
Comparison of icterus in antepartum vs postpartum stage

Mother was vitally stable after delivery, but still had complaints of pruritis, the amount of bleeding was minimal. The values for serum bilirubin were in a decreasing trend post-delivery (Table 1). Ursodeoxycholic acid was maintained after delivery in addition to the preventive antibiotic. The level of icterus also declined during postpartum (Figure 1).

## Discussion

IHCP if detected at a late gestational age, medical care is necessary for this ailment's management and excellent prognosis. The symptoms of ICP have a considerable impact on the patient's quality of life, the challenging part includes diagnosing the illness at the right time. ICP does not warrant an early termination of pregnancy as compared to other liver illnesses [1]. In the COVID era, it was observed that even mild or undetected COVID infection may worsen IHCP.

Itching is one of the primary symptoms of IHCP and often appears in the third trimester [6]. Itching may go missed or neglected because itching is a fairly common symptom. Along with nausea, fecal acolism, vomiting, steatorrhea, and choluria, with jaundice due to fat malabsorption within one to four weeks following the beginning of the pruritus. Prompt and early diagnosis of IHCP can prevent the incidence of respiratory distress in neonates, preterm births, meconium aspiration, and intrauterine deaths [7]. The diagnosis of IHC is challenging due to its low prevalence. Vitamin K deficiency is also associated with intrahepatic cholestasis of pregnancy [8].

In addition to ruling out skin conditions, allergic reactions, liver damage that occurred prior to becoming pregnant, as well as liver damage brought on by numerous other factors not associated with the pregnancy process, such as viral, acute, toxic, or autoimmune hepatitis, the differential diagnosis of additional liver disorders can be made in the clinical context associated with laboratory tests. It is important to diagnose liver disease during pregnancy since early diagnosis can improve maternal and fetal outcomes and decrease morbidity and mortality [6].

Hepatic histology, electron microscopy, liver immunostaining, biliary lipid analysis, molecular analysis, and PMRS spectroscopy are some of the more recent diagnostic methods. In order to avoid pre-term birth and reduce risks to both the fetus and the mother, the objective of treatment is to lower bile acids. Induction at completed 37 weeks should be aimed as there are chances of increased risk of perinatal mortality. Ursodeoxycholic acid treatment in a woman with SARS-CoV-2 infection complicated by intrahepatic cholestasis of pregnancy appears to be associated with a rapid resolution of symptoms and liver function. The administration of obstetric surveillance includes the following actions: weekly liver function test evaluation; weekly fetal cardiotocographic (CTG) starting at 34 weeks of gestation; control of prothrombin time.

## Conclusions

Pregnancy-related intrahepatic cholestasis is a genetic condition. Women who present with pruritus and elevated serum bile acid levels are diagnosed with it. In later life, affected women are more likely to have hepatobiliary problems, but typically only experience transitory gestational cholestasis. Because there is growing evidence that greater levels of bile acid are linked to an increased risk of severe perinatal outcomes, such as stillbirth, bile acid levels should be monitored during pregnancy.

The simultaneous COVID-19 infection with IHCP was managed with strict oxygen saturation monitoring with temperature charting, this mild case did not require any extra treatment for COVID-19 other than monitoring for signs and symptoms. Though the COVID-19 infection was mild, it hampered the delivery of management due to isolation protocols and caused anxiety to our patients. It has been observed that COVID-19 hampers liver enzymes when associated with IHCP. Ursodeoxycholic acid is frequently used to treat intrahepatic cholestasis in pregnancy, which lessens maternal itching and enhances liver function. Active management strategies, which attempt to lower the risk of late stillbirth, are frequently used in conjunction with greater prenatal monitoring and early elective delivery. There is currently insufficient data to support the claim that ursodeoxycholic acid improves the perinatal outcomes or long-term health of pregnant women with intrahepatic cholestasis and their offspring. Obstetricians will have access to much-needed information on which to make treatment choices if future research can overcome these problems.
